# Characteristic Differences between Normotensive and Hypertensive Pseudoexfoliative Glaucoma

**DOI:** 10.3390/jcm13041078

**Published:** 2024-02-14

**Authors:** Da Young Shin, Chan Kee Park, Na Young Lee

**Affiliations:** 1 Department of Ophthalmology, Eunpyeong St. Mary’s Hospital, College of Medicine, The Catholic University of Korea, Seoul 03312, Republic of Korea; eye-sdy1107@hanmail.net; 2 Department of Ophthalmology, Seoul St. Mary’s Hospital, College of Medicine, The Catholic University of Korea, Seoul 06591, Republic of Korea; ckpark@catholic.ac.kr

**Keywords:** normotensive pseudoexfoliative glaucoma, hypertensive pseudoexfoliative glaucoma, lamina cribrosa, central corneal thickness

## Abstract

Purpose: To compare the differences between eyes with pseudoexfoliative glaucoma (PXG) when they are divided into two groups (hypertensive PXG and normotensive PXG) according to the intraocular pressure (IOP). Methods: This is a retrospective study. Data from 86 hypertensive PXG eyes and 80 normotensive PXG eyes were included. Hypertensive PXG was defined as PXG with IOP ≥ 22 mmHg, and normotensive PXG was defined as with IOP ≤ 21 mmHg). Central corneal thickness (CCT) was measured by ultrasound pachymetry. Lamina cribrosa thickness (LT) was evaluated using swept-source optical coherence tomography. Results: No significant differences were observed between hypertensive and normotensive PXG in terms of age, gender, axial length, hypertension, or diabetes. Normotensive PXG eyes had thinner CCT than hypertensive PXG eyes (*p* = 0.02). To compare LT, a sub-analysis was performed after matching age, VF MD and retinal nerve fiber layer thickness. The normotensive PXG group (*n* = 32) demonstrated significantly thinner LT compared with the hypertensive PXG group (*n* = 32) at similar ages and levels of glaucoma severity (*p* < 0.001). Conclusions: Eyes with normotensive PXG demonstrated thinner CCT and LT compared with those with hypertensive PXG, suggesting structural vulnerability to glaucoma.

## 1. Introduction

Pseudoexfoliation syndrome (PXS) is a systemic disease characterized by the gradual deposition of abnormal extracellular materials on various ocular and extraocular tissues [1,2,3,4]. Ocular pseudoexfoliation (PEX) is a well-established risk factor for the development and progression of glaucoma. Pseudoexfoliation glaucoma (PXG) is considered particularly challenging to manage, with a high treatment failure rate. Although it is typically associated with elevated intraocular pressure (IOP), PXG has been reported to exhibit a more serious clinical course and a worse prognosis than primary open-angle glaucoma (POAG) with a similar level of IOP [5,6,7,8]. This suggests that PEX itself could be a risk factor for glaucoma, independent of the contributory effect of raised IOP.

In clinical practice, it is not uncommon to observe patients with PXG whose IOP never exceeds 21 mmHg. Several authors have documented such cases. For instance, Yarangumeli et al. [9] and Puska et al. [10] reported instances of glaucomatous damage in the normotensive fellow eyes of patients with unilateral PXG. Koz et al. [11] presented findings on the prevalence of normotensive glaucoma in PXS. Rao [12] documented five eyes with normotensive PXG. Despite these reports highlighting eyes with PXG and normal IOP, comprehensive comparisons between normotensive PXG and hypertensive PXG have not been well-documented.

The aim of the present study was to compare PXG in two groups based on IOP levels and investigate the differences in various ocular parameters. We assessed the characteristics of the lamina cribrosa in PXG patients using swept-source OCT (SS-OCT).

## 2. Methods

### 2.1. Patients

This is a retrospective, comparative, cross-sectional study. All study subjects were examined at the glaucoma clinic (Dr. Park) of the University of Seoul St. Mary’s Hospital between 1 January and 31 December 2019. Each subject underwent a complete ophthalmologic examination, which included slit lamp biomicroscopy, IOP measurement using Goldmann applanation tonometry, central corneal thickness (CCT) assessment with ultrasound pachymetry (UD-800, Tomey Corporation, Nagoya, Japan), axial length measurements using ocular biometry (IOL Master; Carl Zeiss Meditec, Dublin, CA, USA), dilated stereoscopic examination of the optic disc, red-free fundus photography (Canon, Tokyo, Japan), Cirrus OCT (Carl Zeiss Meditec, Jena, Germany) and a visual field (VF) test on a Humphrey VF analyzer employing the Swedish Interactive Threshold Standard 24-2 algorithm (Carl Zeiss Meditec, Dublin, CA, USA). 

The inclusion criteria of this study were as follows: presence of PEX material in the anterior chamber and evidence of glaucomatous damage. The exclusion criteria included a history of other optic nerve diseases, retinal diseases or ocular trauma. One eye was selected randomly in cases where both eyes fulfilled the inclusion criteria.

All eligible participants were included in the chart review. All procedures adhered to the principles of the Declaration of Helsinki. As the data were retrospectively evaluated pseudonymously and were solely obtained for treatment purposes, the requirement for informed consent was waived by the Ethics Committee of Seoul St. Mary’s Hospital, Korea (Ethical approval no. KC21RISI0751, 8 October 2021). 

### 2.2. PXG

The presence of PEX material and evidence of glaucomatous damage defined PXG. PEX material, characterized by white, fluffy, fibrillar tissue, was confirmed through slit lamp biomicroscopy of the anterior capsule or pupillary margin after pupillary dilatation. Glaucomatous damage was identified by the presence of glaucomatous optic nerve damage, such as diffuse or focal neuroretinal rim thinning or an enlarged vertical cup-to-disc ratio, along with an associated VF defect. Glaucomatous VFs were defined by meeting two or more of the following criteria: (1) a cluster of three or more non-edge points on pattern deviation plots with a probability of <5% of the normal population, with at least one of these having a probability of <1% or a cluster of two or more non-edge points on pattern deviation plots with a probability of <1%; and (2) glaucoma hemifield test results outside the normal limits; and (3) pattern standard deviation of less than 5%. 

IOP was measured with a Goldmann tonometer, and the corrected IOP was calculated using the following formula to correct errors due to corneal thickness [13].

Corrected IOP = Measured IOP − (CCT − 545)/50 × 2.5 mm Hg

The peak IOP represented the highest IOP value recorded during follow-up, while the mean IOP was calculated as the average of all measurements taken within the 3 years preceding the last visit. 

### 2.3. Hypertensive and Normotensive PXG

Hypertensive PXG was defined as an IOP > 21 mmHg before or after treatment. Normotensive PXG was defined as an IOP ≤ 21 mmHg both before and after treatment, Normotensive patients had no recorded IOP > 21 mm Hg. The diagnosis of both types of glaucoma was based on uncorrected IOP criteria.

### 2.4. Posterior Pole Profile

These values were measured using image analysis software (ImageJ version 1.40; developed by Wayne Rasband; available at http://rsb.info.nih.gov/ij/index.html, accessed on 18 January 2024) on disc fundus photographic image.

The technique for assessing the posterior pole profile has been previously described and applied in earlier investigations [14]. Optic disc tilt was defined as the ratio between the longest and shortest diameters of the optic disc [14,15,16]. Optic disc torsion was identified and defined as the deviation of the long axis of the optic disc from the vertical meridian. The disc foveo angle was determined as the angle between the line connecting the fovea and the center of the disc and the horizontal line passing through the center of the disc [14,16,17]. This measurement was automatically conducted using the Heidelberg SPECTRALIS OCT (SPECTRALIS software v. 5.1.1.0, Eye Explorer Software 1.6.1.0; Heidelberg Engineering, Heidelberg, Germany). Peripapillary atrophy (PPA) and disc area were measured through optic disc photography, with pixel areas calculated using ImageJ software version 1.40 [14]. For ease of evaluation, a PPA/disc ratio of 0.4 was utilized as the cut-off value to assess the stress level of PPA on the optic disc. This value was referenced from a prior study investigating the beta zone PPA/disc ratio in glaucoma [18].

### 2.5. Laminar Cribrosa Assessment by SS-OCT

**Figure 1:**
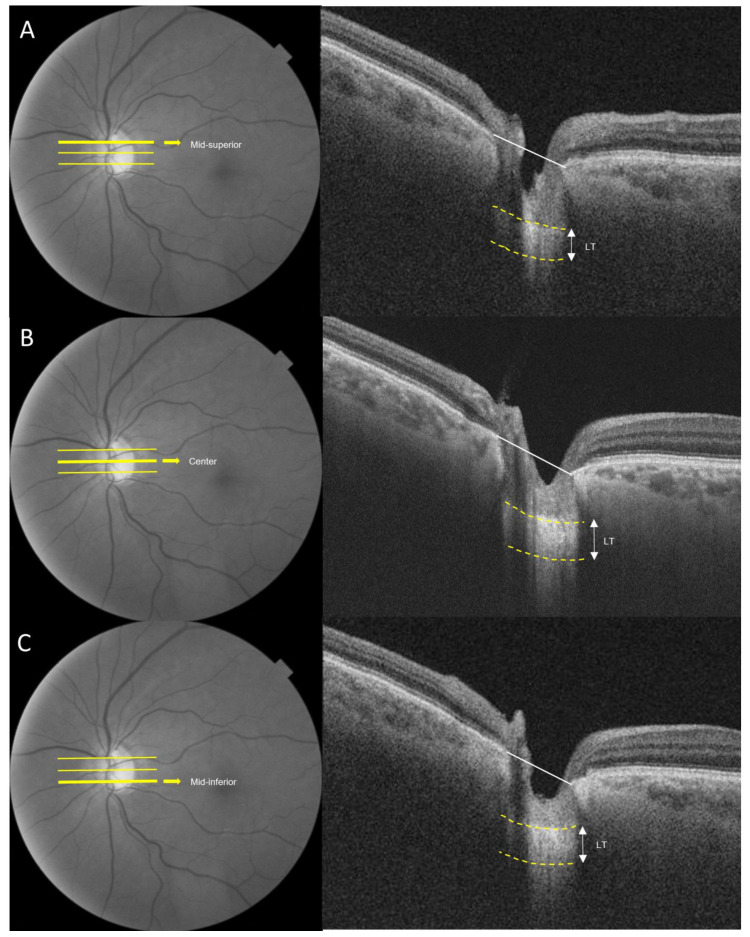
Measurement of the lamina cribrosa using SS-OCT. The anterior and posterior borders of the lamina cribrosa are indicated by the yellow dotted lines. Lamina thickness (LT) was defined as the distance between the two yellow dotted lines. LT was estimated from the three B-scans (mid-superior, center, mid-inferior) (**A**) Mid-superior. (**B**) Center. (**C**) Mid-inferior. The average of the three measurements obtained from the three points was considered the representative value of the LT.

Images of the lamina cribrosa were acquired using an SS-OCT device (DRI OCT Triton; Topcon Corporation, Tokyo, Japan). SS-OCT was conducted at a scan speed of 100,000 A-scan/s and had a long wavelength that is centered at 1050 nm, surpassing the conventional Spectral domain OCT (840 nm). SS-OCT provided an approximately 8 μm axial resolution, enhancing the imaging of deep ocular structures. LT was assessed at three locations (mid-superior, center and mid-inferior) that passed through the optic nerve head. LT was measured as the distance between the anterior and posterior borders of the highly reflective region beneath the optic nerve head in a cross-sectional B-scan (Figure 1) [19,20]. Adobe Photoshop (Version 7.0, Adobe Systems, Inc., San Jose, CA, USA) was utilized to assess the anterior and posterior borders. The LT was measured using the manual caliper tool in DRI OCT Viewer (Topcon, Tokyo, Japan). Two observers (D.Y., N.Y.) independently measured LT, and each measurement was repeated twice in every B-scan. The mean of the four measurements was reported as the LT in each B-scan. To assess the intra-observer and inter-observer reproducibility of LT measurements, 25 randomly selected SS-OCT cross-sectional B-scans were evaluated, and correlation coefficients were calculated for inter-observer and intra-observer variability.

### 2.6. Statistical Analysis

Sample size calculations were conducted using G*Power 3.1 software, resulting in a minimum sample size of 128 total, with an effect size set at 0.5 (minimum size), an alpha error of 0.05, and a power of 0.8. Data are presented as means and standard deviations. Independent Student *t*-tests were employed to compare data between the two groups (normotensive PXG and hypertensive PXG), while Chi-squared tests were used for categorical variables. A significance level of *p* < 0.05 was considered statistically significant. All statistical analyses were performed using SPSS for Windows (Version 24.0; IBM Corporation, Armonk, NY, USA).

## 3. Results

A total of 166 eyes with PXG were included in the study, comprising 80 normotensive PXG eyes and 86 hypertensive PXG eyes. The average observation period for PXG patients was 5.48 ± 3.50 years. Table 1 presents comparisons between normotensive and hypertensive PXG. No significant differences were observed in terms of age, axial length, hypertension or diabetes. The hypertensive PXG group had a higher percentage of males (61.6%), while the normotensive PXG group had a higher percentage of females (66.3%) (*p* < 0.001). The average IOP was 17.95 ± 6.60 in hypertensive PXG and 14.30 ± 2.95 in normotensive PXG (*p* < 0.001). The corrected average IOP was 18.22 ± 6.95 in hypertensive PXG and 15.17 ± 3.37 in normotensive PXG (*p* = 0.001). Normotensive PXG eyes exhibited thinner CCT (523.30 ± 32.15 µm) compared to hypertensive PXG eyes (544.71 ± 61.84 µm, *p* = 0.02). There were no statistical differences in disc tilt, disc torsion, disc fovea angle or PPA to disc ratio (>0.4) between the two groups. Eyes with hypertensive PXG showed more severe glaucomatous damage than those with normotensive PXG.

**Figure 2:**
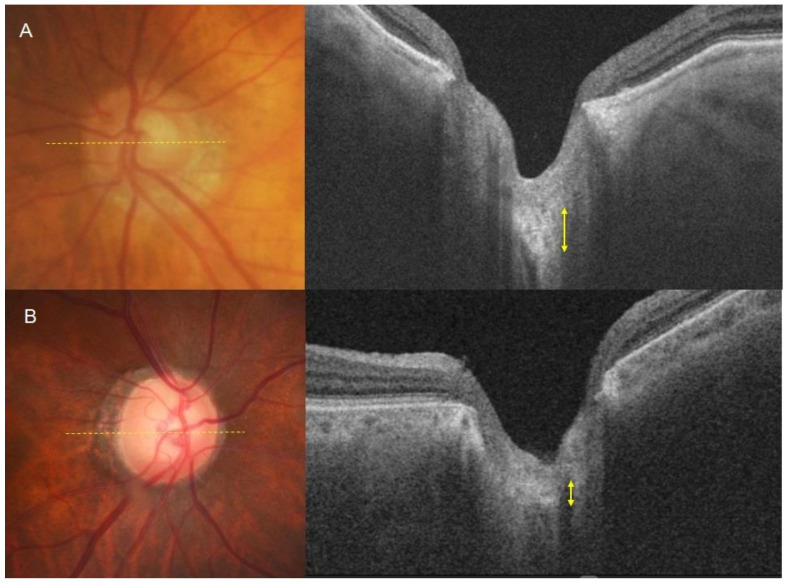
Measurement of the lamina cribrosa using SS-OCT. (**A**) An 80-year-old man with hypertensive pseudoexfoliative glaucoma exhibited a thick lamina cribrosa. The axial length of this eye is 22.79 mm, and the MD of the visual field test is −10.63 dB. (**B**) A 77-year-old woman with normotensive pseudoexfoliative glaucoma displayed a relatively thin lamina cribrosa. The axial length of this eye is 22.43 mm, and the MD of the visual field test is −11.28 dB.

In a comparison of age- and disease-matched eyes between the two groups, the normotensive PXG group showed significantly thinner LT in the mid-superior and mid-inferior regions. The intra-observer and inter-observer correlation coefficients were 0.906 and 0.929, respectively. The mean LT was 178.61 ± 15.74 for the hypertensive PXG group and 159.84 ± 15.60 for the normotensive PXG group (*p* < 0.001, Table 2). Figure 2 illustrates representative cases from the hypertensive PXG and normotensive PXG groups. For instance, an 80-year-old man with hypertensive PXG exhibited a thick LC with VF MD −10.63 dB (axial length 22.79 mm). In contrast, a 77-year-old woman with normotensive PXG displayed a relatively thin LC, with VF MD −11.28 dB (axial length 22.43 mm).

## 4. Discussion

This study investigated the differences in characteristics between normotensive and hypertensive PXG. The normotensive PXG eyes had thinner CCT than hypertensive PXG eyes. The lamina cribrosa was also significantly thinner in normotensive PXG eyes than in hypertensive PXG eyes with similar severity of glaucoma.

PXS is a systemic disorder that has ocular manifestations. PEX is a fibrillar material that contains components of the elastic fiber and basement membrane system. It is unclear whether PEX is due to excessive synthesis or inadequate breakdown of that component. The accumulation of PEX is likely the result of an imbalance between synthesis and degradation. PEX material can block the trabecular meshwork and cause trabecular cell dysfunction. Not all patients with PXS have glaucoma, but when glaucoma develops in PXS, it generally manifests as high IOP and large IOP fluctuation [3,4]. Therefore, PXG is thought to have a poor prognosis, rapid progression, poorer response to medications, and an increased need for surgery [5,6,7,8]. Because of this clinical significance, many studies of PXG have been conducted. A number of previous studies have already demonstrated that PEX itself could be a risk factor for glaucoma, without the contributory effect of raised IOP [9,10,11,12]. Vasculopathy and lamina cribrosa weakness have been suggested as IOP-independent mechanisms for the development or progression of glaucoma. Furthermore, we reported an increased risk for glaucoma in the non-PEX fellow eye [21]. In that study, the retinal nerve fiber loss was faster in normal tension glaucoma (NTG) eyes with PXS than in control NTG eyes [21].

Although the importance of IOP-dependent mechanisms has been increasingly highlighted, most studies have concentrated on PXG with high IOP. However, we have frequently seen cases of PXG with IOP not exceeding 21 mmHg during the observation period. In fact, there have been several reports about PXG with normal IOP dating from several decades ago. These reports included normotensive eyes with PEX material that had optic disc changes [10], VF defects [22] and glaucomatous optic neuropathy [9,11,12].

However, no study has investigated the characteristics of PXG according to IOP level. Open-angle glaucoma can be divided into POAG and NTG based on IOP. Differences between POAG and NTG are well-established in terms of clinical characteristics and pathogenesis. It has been reported that the lamina cribrosa is thinner in NTG than in POAG, and blood flow is more significantly impaired in NTG than in POAG [23,24]. NTG is more associated with peripapillary scleral deformation, such as tilt ratio, torsion degree and PPA. Posterior pole deformation often explains the susceptibility to glaucomatous damage in NTG [17,25,26]. These findings prompted us to ask whether there are similar differences between normotensive and hypertensive PXG.

Various ocular parameters were compared in normotensive PXG and hypertensive PXG to evaluate this. Axial length, CCT, disc tilt, torsion, disc fovea angle, PPA/disc ratio and LT were included. The lamina cribrosa is associated with glaucoma severity. It is deformed according to glaucomatous damage. Age is also a risk factor for glaucoma and PEX. To reduce possible bias, we conducted a case-control study by selecting 64 patients matched in age and VF MD (1:1 matched; 32 normotensive PXG and 32 hypertensive PXG) (Table 2).

The lamina cribrosa is the sieve-like structure of the optic nerve head, which supports the unmyelinated retinal ganglion cell axons [27,28]. Deformation and displacement of the lamina cribrosa have long been identified as the main pathophysiologic mechanisms of glaucomatous optic neuropathy [27,28,29]. PXS has been strongly associated with single nucleotide polymorphisms of the lysyl oxidase-like 1 (LOXL1) gene, which is one of a group of enzymes involved in the crosslinking of collagen and elastin in the ECM [30,31]. The proliferation and degeneration of elastic elements, termed elastosis, is manifested ultrastructurally by increased production of elastic microfibrils, large bizarre elastic fibers, atypical aggregation and abnormal surrounding matrix [32]. The lamina cribrosa is composed of collagen fibers and elastin [33,34]. Therefore, abnormal elastosis may also weaken the lamina cribrosa in PXS [35,36,37]. An imaging study using OCT demonstrated that the lamina cribrosa in PXG is thinner than that in POAG [38]. These features of the lamina cribrosa were observed not only in PXG eyes but also in the apparently normal-looking fellow eye [38]. Histological study showed that the stiffness of the lamina cribrosa was lower in PXS than in controls [35]. Marked and widespread elastosis in the connective tissue of the lamina cribrosa of eyes with PXS was confirmed by electron microscopy [35]. Elastosis of the ECM in the lamina cribrosa could initiate or increase susceptibility to elevated IOP, leading to optic nerve damage in patients with PEX [35]. That might explain the difference between normotensive and hypertensive PXG. We also demonstrated that the LT differed between hypertensive and normotensive PXG, as it does between POAG and NTG.

Thin CCT is a well-known risk factor for the development of glaucoma [39,40,41]. Because Goldmann applanation tonometer measurements are ultimately dependent on CCT, whether CCT is truly an independent risk factor for glaucoma has remained an unanswered question. Many previous studies have shown that NTG is associated with thinner CCT, compared with POAG [42,43,44]. Our study showed that normotensive PXG eyes (523.30 ± 32.15 µm) have thinner CCT than hypertensive PXG eyes (544.71 ± 61.84 µm, *p* = 0.02). Since IOP values were used when classifying eyes into the two groups, this difference may be a predictable result rather than a pure difference in characteristics. In our study, the average CCT difference between the two groups was 21.41 µm, raising the CCT-corrected IOP by about 1–2 mmHg. A previous study reported that CCT remained a statistically significant predictor in multivariate models, including CCT-corrected IOP [45]. There are several possible explanations other than the effect of the IOP measurement error. CCT is a biomarker for weaker biomechanical properties involved in the pathogenesis of glaucoma, such as the sclera or lamina cribrosa [46]. Thinner corneas correlated with the response of the corneoscleral shell and the ocular vasculature to IOP-induced stress [47]. A recent study demonstrated thinner CCT is associated with a decreased parapapillary vessel density in NTG [48].

Our study showed that hypertensive PXG was associated with the male gender, and normotensive PXG was associated with the female (*p* < 0.001, Table 2). Published studies on PXS are inconsistent regarding gender differences; although some previous studies reported a higher prevalence among women than men, many other studies found no gender difference [49,50,51,52]. Gender associations are not always reproducible, and this discrepancy in gender susceptibility has not yet been explained.

In our study, there was a statistically significant difference in the prevalence of pseudophakia between the two groups. Patients with hypertensive PXG not only underwent cataract surgery more frequently than those with normotensive PXG but also had a higher incidence of glaucoma surgery. Considering the tendency for cataract progression to accelerate after intraocular surgery, it can be inferred that cataract surgery is typically performed earlier in patients with hypertensive PXG [53].

Our study had some limitations. First, it had a relatively small sample size. There was a relatively small portion of patients with advanced glaucoma. We did not include glaucoma eyes without a DH. Further study is needed to confirm our findings in patients with advanced glaucoma. Second, it is difficult to generalize our findings because only Korean patients were studied. Since most Korean glaucoma patients are normal-tension glaucoma (NTG) and our study subjects also had untreated IOP in the range of NTG, our finding could be considered as characteristics of NTG patients with DH. 

Here, we reported differences between hypertensive PXG and normotensive PXG. To our knowledge, this is the first study to divide PXG eyes into groups according to IOP level. This study highlights that some PXG have consistently normal IOP, and our results suggest that the mechanism underlying glaucomatous damage may be different in the two groups.

## Figures and Tables

**Table 1:** Differences in demographic and clinical characteristics between hypertensive and normotensive pseudoexfoliative glaucoma.

Variable	Hypertensive PXG (86 Eyes)	Normotensive PXG (80 Eyes)	*p*-Value
Age, year	73.78 ± 8.41	73.58 ± 10.08	0.890
Sex, M/F	53/33	28/53	**<0.001**
CCT, μm	544.71 ± 61.84	523.30 ± 32.15	**0.021**
Axial length, mm	23.70 ± 1.04	23.85 ± 1.24	0.451
Hypertension, *n* (%)	35 (40.7%)	28 (34.6%)	0.414
Diabetes, *n* (%)	14(13.3%)	8 (9.9%)	0.200
Pseudophakia, *n*	37 (44.0%)	23 (28.4%)	**0.037**
Medication eyedrop	1.63 ± 0.75	1.93 ± 1.02	**0.032**
Glaucoma surgery history	52 (60.5%)	4 (4.9%)	**<0.001**
Mean IOP, mmHg	17.95 ± 6.60	14.30 ± 2.95	**<0.001**
Corrected mean IOP, mmHg	18.22 ± 6.95	15.17 ± 3.37	**0.001**
Peak IOP, mmHg	30.68 ± 8.25	17.78 ± 2.82	**<0.001**
Corrected peak IOP, mmHg	30.84 ± 8.33	18.53 ± 3.27	**<0.001**
RNFL thickness, μm	59.08 ± 11.75	66.74 ± 9.84	**<0.001**
VF MD, dB	−17.59 ± 10.91	−8.69 ± 8.32	**<0.001**
Follow-up period, year	5.69 ± 3.81	5.26 ± 3.13	0.470
Tilt ratio	1.12 ± 0.94	1.14 ± 0.12	0.361
Torsion ratio	90.73 ± 6.96	89.44 ± 8.63	0.611
Disc fovea angle	−6.68 ± 5.36	−6.20 ± 4.06	0.563
PPA/Disc area > 0.4	18 (20.5%)	20 (25.0%)	0.533

CCT—central corneal thickness; IOP—intraocular pressure; VF—visual field; MD—mean deviation; RNFL—retinal nerve fiber layer. Statistically significant values appear in bold. Continuous data are mean ± mean standard deviation unless otherwise indicated.

**Table 2:** Comparison of lamina cribrosa thickness between eyes with hypertensive and normotensive pseudoexfoliative glaucoma matched for patient age, visual field mean deviation and retinal nerve fiber layer thickness.

Variable	Hypertensive PXG (32 Eyes)	Normotensive PXG (32 Eyes)	*p* Value
Age, year	74.78 ± 8.91	75.22 ± 7.99	0.837
Axial length, mm	23.47± 0.81	23.68 ± 1.02	0.434
RNFL thickness, μm	68.13 ± 11.45	69.00 ± 10.76	0.754
VF MD, dB	−9.02 ± 8.78	−8.92 ± 8.71	0.962
VF PSD, dB	5.68 ± 4.14	6.01 ± 3.48	0.737
**Lamina Cribrosa Thickness**			
Mid-superior, μm	176.16 ± 22.74	151.66 ± 20.88	**<0.001**
Center, μm	188.50 ± 29.21	176.63 ± 24.70	0.084
Mid-inferior, μm	171.19 ± 31.70	151.25 ± 22.31	**0.005**
Mean, μm	178.61 ± 15.74	159.84 ± 15.60	**<0.001**

VF—visual field; MD—mean deviation; RNFL—retinal nerve fiber layer. Statistically significant values appear in bold. Continuous data are mean ± mean standard deviation unless otherwise indicated.

## Data Availability

The data presented in this study are available on request from the corresponding author. The data are not publicly available due to ethical reasons.
